# Philadelphia Hospital

**Published:** 1838-02-28

**Authors:** 


### PHILADELPHIA HOSPITAL.

### NEURALGIA.

(*Concluded from page* 70.)

*Saturday, January 27th.*—(Dr. Jackson continued.) I shall now mention briefly to you the causes of neuralgia. These are of various characters. Temperament has a decided influence; a nervous, or a nervo-sanguine temperament particularly predisposes to this form of disease. A sedentary mode of life is very often a cause of neuralgia, perhaps from the disorders induced in the digestive function. Particular habits of diet give origin to it. A poor diet, debilitating the individual, and impoverishing the blood of its red-globules and colouring matter, is a common cause of this affection. An abstinence from generous wine and stimulating liquors, where these are demanded by the enfeebled state of the digestive forces, may be mentioned also as predisposing causes. Previous attacks of disease, leaving the patient in a feeble and weakened condition, or in which the nervous organs have suffered, very often are succeeded by intractable neuralgias. This is especially the case with intermittent fevers; so common indeed is neuralgia after that affection, that McCullough in his work on Malaria, regards the two diseases as identical. All neuralgic affections, he asserts, are concealed intermittents. This, I think, is too exclusive; it is pushing a favourite notion to too great an extent. The influence of malaria in the production of neuralgia is undoubted. We continually meet cases of neuralgia in persons who live in malarial districts, and in those who have suffered from intermittent fever, and it often assumes the intermittent type so completely as to identify the two diseases. But to say that neuralgia in all its forms is always caused by malaria, seems to me entirely too exclusive a doctrine. The predisposing causes of this disease are innumerable; whatever indeed tends to weaken down the forces of the individual, exhaust or impoverish the circulating fluid, and impair or vitiate nutrition, predisposes to this variety of disease. The proximate cause belongs to the nervous organs of sensibility, and consists in the morbid production, or perversion of this functional agent. The predisposing causes, however diversified, appear to have this termination.

When this condition of sensibility is once produced, the exciting causes of an attack may consist in any thing that may make an unusual and decided impression on the system, disturbing thereby the state of the economy, or deranging its functions. Hence it is that the exciting causes are most diversified in character.

The weather, sudden alternations of heat and cold, electric changes of the atmosphere, north-easterly winds, in this country are often productive of attacks. They are induced, too, by loss of rest; prolonged exertion of mind and body; disturbing moral and mental emotions; anxiety and distress of mind. Indigestable food, acid wines or drinks, acid generally in the stomach from indigestion, and constipation of the bowels are among the most frequent excitants of an attack. Indurated fecal matter, or even a lodgment of fecal matter in the rectum for a few hours, is sufficient to excite neuralgic pains. It is in such cases necessary to observe the greatest vigilance in this respect, and to obtain one or two movements daily. This is exemplified in my own case. If the bowels are sluggish, every morning I wake with neuralgic pains of the lower extremities, which pass off as soon as a movement has taken place. When the exciting cause is in the rectum, the lower extremities are the parts in which neuralgia is developed. When disorder of the stomach, or upper bowels, give origin to the paroxysm, it is the chest, head, or abdomen in which the pain is seated.

In females a most common exciting cause is uterine disorders, as prolapsus, inflammation of the neck of the womb, and more particularly dysmennorhœa. It is a difficult point to determine in many cases, whether this latter affection is not neuralgia itself, the uterus being the seat of the neuralgic pain, and affected primarily. I have repeatedly known cases where the health has been unimpaired until the time for the appearance of the catamenia, when a general affection was developed, preceded by local uterine suffering. I am now attending a case of this kind. The patient has been suffering under the various forms of neuralgia for the last ten years. They commenced with dysmennorhœa. She has been treated for hepatic, and other supposed diseases ineffectually, the true character of the disorder having been overlooked, until the constitution is completely broken down. In many instances the nervous disorder is not confined to the production of neuralgia, but a general neuropathy is induced, every function of the nervous apparatus, cerebro-spinal and ganglionary becoming involved.

A lady from Virginia some years past was a patient of mine, in whom this universal neuropathy occurred. She suffered with neuralgic pains rapidly travelling throughout her frame. The muscular system was tormented with irregular spasmodic contractions, by which her body and limbs would be fixed for hours in distorted postures the most painful. The stomach, bowels, muscles of the pharynx and larynx, and the heart were frequently attacked with spasm. She had hallucination and perversion of the senses—double consciousness, two minds, as it were, alternately acting. The internal organs were equally disordered. The stomach did not digest, the bowels were constipated, the urine sometimes was suppressed, and vicariously discharged from the rectum. This case yielded after some months on the restoration of the uterine functions. There are constantly existing in this city similar cases amongst young females, some almost I have known quite rivalling the above in the intensity and variety of the nervous disorders, and connected more or less with derangement of the uterus.

I might occupy your time by enumerating a vast variety of causes; but this would be unnecessary. It may briefly be said, that whatever will produce any uncommon sensation or impression on any of the organs, may prove an exciting cause of an attack of neuralgia in those predisposed to the disease. We are not then to look at the exciting cause, or to the organ in which its action is developed as the seat of the disease, but we are to direct our attention to the nervous centres of sensibility as the proper seat of neuralgia, the excessive or perverted functions of which we are to rectify.

The treatment of this affection will now occupy our attention. In functional neuralgia whatever tends to diminish the quantity and quality of the blood, predisposes to the disease. There appears to exist a sort of antagonism between nervous activity, and the quantity of plastic material, red globules, and colouring matter in the blood. The nervous and sanguine systems appear to balance each other. When the blood becomes impoverished, when there exists a want of a sufficient quantity of red globules, we are, I told you, as a consequence of this anemic condition, very apt to have neuralgia developed. This is not a necessary consequence of this condition of the blood; it does not invariably happen. Whatever then tends to weaken the system, and impair the nutritive function, as antiphlogistic treatment, is improper in this disease. The drastic purgatives are exceedingly pernicious, and very often bring on an attack. The quack pills, which are generally of a violent drastic character, very often do this. I had a young lady under my charge some time since, who being constipated had recourse to Morrison's pills. They relieved her, and she was so much pleased with them that she continued on their use, increasing the quantity as indicated in the directions. The consequence was a violent cholera morbus, which left her in a prostrated condition; the digestive functions became impaired, and general neuralgia attacked her soon afterwards, from which she recovered only after three years of every variety of suffering. After the prevalence of the cholera in this city, neuralgia, especially of the internal organs, was very common, and serious mistakes were often made in the treatment. Since that epoch, it appears to me that neuralgic diseases have increased greatly. In your treatment of neuralgia, the antiphlogistic and debilitating treatment is to be employed with great reserve, and only when some spinal irritation exists. Our main reliance must be placed on the tonics, and on an alterative treatment. These remedies are to be conjoined with the narcotic and sudorifics; these last articles will aid materially the treatment. Whenever warmth, softness and moisture of the skin is produced, the disease is mitigated. The objects to be aimed at in the treatment are to invigorate the system and expend and equalize nervous excitement by producing a movement of diffusion—of expansion by diffusibles and sudorifics. The case of the lady which I have just mentioned, apparently of so desperate a character, got well under this method of treatment. To the remedial means calculated to accomplish this irradiation to the surface, must be conjoined good diet, and the tonics. This unfortunately cannot always be done. The digestive forces are sometimes so completely suspended that we cannot resort to these measures. This is particularly the case with the internal neuralgias, and those cases of general neuropathy. A patient whom I am now attending, presents this difficulty. There is total absence of appetite, even disgust for food, and when it is taken, it invariably disagrees with the stomach. Patients in this form of the disease will pass whole days taking no other aliment than water and some bread. This state will continue for months, and I have known it in two cases to last for two years. What is extraordinary is, that emaciation does not take place under this abstinence. On the contrary, the embonpoint continues as perfect as in health.

In regulating the diet, we should be especially careful to prevent our neuralgic patients from indulging in those stimulants, which, while they excite the nervous system, do not afford any nutrition, such as coffee, tea, &c. &c. A rigid abstinence from such articles should be observed.

The bowels should be kept in a soluble state, but active purging I have never found useful. Drastics often occasion severe pain in the bowels, and render the pain of the back and other organs worse.

We are repeatedly called upon during an attack to relieve the sufferings the patient is enduring. The narcotics are in the first rank of remedies for the palliative treatment. The use of opium is I believe injurious, and should not be resorted to except in very violent cases. This narcotic deranges more than any of the others the digestive functions, and we have very serious consequences resulting from its constant employment. I much prefer belladonna, aconite and veratria.

If we were to credit the accounts which are given by Turnbull of the efficacy of this latter article, neuralgia would cease to be any longer an object of dread or reproach to the practitioner. We should possess a remedy which would soon dissipate every vestige of the disease. Unhappily, experience does not realise our expectations. I have tried it repeatedly, and have been much disappointed with it. I do not think that it relieves more than three or four times in twelve. This article appears to exercise some specific influence over sensibility. It diminishes to a certain extent sensation; it paralyses, as it were, the sensibility of the part to which it is applied; its application produces very much the sensation which we experience in a limb said to be asleep. It certainly does in neuralgic paroxysms cause a diminution of pain. In the neuralgia about the eye and face, depending on a branch of the fifth pair of nerves, I have, I think, seen it more frequently beneficial than in neuralgia of the extremity. Where the pain is fixed and violent, in no one case have I seen the least advantage result from its employment. In my own case, which is wandering neuralgia, I have repeatedly used it, but without the slightest utility. In one case under my care, I derived much benefit from it. Here, along with general neuropathy, neuralgic pains were severe. The integuments of the back were so sensitive, that I could not touch it with my finger, and I was thus precluded from the employment of counter irritation. An ointment of veratria and laurel oil, applied on the back with a feather, gave entire relief to this system. I was then enabled to insert a seton, and employ moxas. The manner in which I use veratria, is to mix it up with laurel oil, with which it incorporates very readily, and forms with it a soft unguent. It is generally combined with simple cerate, but this medium prevents to a certain extent the action of the veratria. It may be administered internally in the form of a pill, in the dose of one-eighth of a grain. From its internal use I have derived advantage, in treating the internal neuralgias when limited in extent. You must be careful in the internal administration of this remedy. It is very active. You must watch your patient closely, or you may have irritation of the stomach and bowels brought on, or a cholera morbus induced. The first symptoms of diarrhœa, heat in the stomach and indigestion, must warn you to desist.

I place great confidence in belladonna and in aconite. This last article has been lately highly recommended in Europe. These may be administered alone, or, what I prefer, is to give them in combination united with some tonic, as the extract of gentian.

The tonics have been also much extolled. When there exists an anemic condition—a deficiency of red blood—the chalybeates are undoubtedly the most efficacious means. When these were first proposed, the most extravagant praise was lavished on them. Hutchinson, who was the first to recommend the carbonate of iron in neuralgia, published a number of very miraculous cures effected by it. In this country it has been found only of limited utility. I have known it to be taken in as large a quantity as a pound a day ineffectually. This was in the case of a gentlemen who had facial neuralgia. The superior maxillary branch of the fifth pair of nerves was affected. The pain was shooting or darting, returning every three to five minutes. He was almost in a state of distraction from his suffering. He took the iron at first in large quantities, but obtaining no relief, he procured a pound, mixed it up with some molasses and took the whole off in the course of twenty-four hours. No good result followed. For myself, I have been much disappointed with the chalybeates, except in those cases which I have indicated depending upon anemia. By improving the quality of the blood, the nervous disorder subsides. In all other cases, they have been useless.

Quinia, I consider a remedy of great value. In the intermittent neuralgia, it is the only one on which we can rely with any certainty, and in that form of the disease, it rarely fails. I always combine with it sulphate of morphia. It must, however, be pushed to a considerable extent, if we anticipate a favourable issue. This, though, sometimes cannot be done with safety, the condition or the internal organs forbids its employment. In the case I mentioned, where an enormous quantity of the carbonate of iron was taken without effect, quinia proved successful. It was given in doses of from grs. x to grs. xv several times a day.

Where you suspect the spine to be the primary seat of the affection, you must direct your attention to it. If you rely on the statements made by Tate, Teale and others, you would be led to believe that pain from pressure on the spine existed invariably in all neuralgic affections. This is far from being the case. It requires a particular condition of the spinal meninges for pain or pressure along the vertebræ to be produced. Nor is the absence of pain a proof of the non-existence of the disease. In such cases, where the patient is of a sanguine temperament, you may employ local depletion with advantage, as cups or leeches along the spine. On the contrary, where anemia exists, you must be cautious how you detract blood by any means.

The use of counter-irritants have been recommended, and much utility will result from their employment. Of the counter-irritants, the tartar emetic ointment has been highly praised. Let me caution you how you employ it. It is very apt to prove excessively painful to the patient, and, in consequence, often aggravates the symptoms. Pain is a direct excitant of the nervous centres, the seat of the disease. I am convinced, that whatever application occasions prolonged suffering in neuralgia, is injurious. The application I prefer, is a liniment composed of croton oil, turpentine, oil of cinnamon, and sweet oil. The use of this is often followed by the happiest results. Its application causes inflammation, accompanied with a pimply eruption along the back, and seldom causes painful feelings.

In the extensive and violent cases recorded, where the neuralgia is general, occupying all the organs, persistent counter-irritants along the spine are then the most effective means to be resorted to. Setons, I think, are to be preferred. No advantage is obtained until they cause profuse suppuration. Two or three are to be inserted in the course of the spine.

I have employed the moxas very often. In the lady from Virginia, I must have used upwards of three hundred moxas at various times. They were applied three times a day for some months. I seldom carrying the moxas to the extent of producing deep eschars; a slight vesication is all I seek to produce, together with external rubefaction by passing the incandescent cone over a considerable surface, holding it a short distance from the surface. Acupuncturation, among other local remedies, has been resorted to. Its effects are not constant. In neuralgia about the eye, I have known it to relieve the patient more frequently than in any other form of the disease. In the fixed, deep-seated pain of the spine, it does not impart relief. Dr. Mitchell informs me that by inserting short gold pins along the spine, and permitting them to remain permanent for some days, that he has succeeded in procuring relief. I have tried this plan in very severe and intractable cases, but it did not succced as I had anticipated. Some years ago, I relieved a case of internal neuralgia that had resisted all treatment for upwards of a year, by acupuncturation. The pain was seated in the cardiac region. I inserted a needle of sufficient length to penetrate into the thorax. The relief was immediate, and, after repeated insertions, the pain finally ceased.

Acupunctuation cannot, however, be relied on with any certainty, the relief it affords being rarely permanent.

Galvanism has been much extolled, and has become quite an empirical remedy. I have employed this agent extensively, but in neuralgic disease cannot speak of it in confident terms. There are some nervous affections—a limited range—in which its effects are prompt and admirable. From the observations which I have made, when it is employed, it is the sensible currents that are most useful. When it is so strong as to give shocks or severe pain, it often aggravates the neuralgic disease, and renders it more obstinate. I have found it also more serviceable in gastralgia and in enteralgia than in other forms. The apparatus I use in such cases, is very simple, a cylinder of zinc, about 3 or 4 inches long and $\frac{1}{4}$ of an inch in circumference, is introduced into the rectum; a silver oval plate is placed in the mouth, and the two are united by a silver wire. This apparatus is to be kept applied for an hour at a time, once or twice daily. It improves the digestive powers, and often obviates obstinate constipation. Galvanism has never relieved neuralgic pain fixed in the spine, or external fixed neuralgic pain, in my experiments with it.

Blisters, sinapisms and other painful remedies, applied so that the painful impression is transmitted through the *pained* nerve, always increases the suffering. On the contrary, soothing applications, camphorated, anodyne, and oleaginous liniments are to be preferred. The evaporating lotion made tepid, is more frequently alleviating, and pleasant as a local application than any other.

The influence of temperature is quite uncertain, and it is impossible to ascertain before hand, whether warmth or cold will prove the most preferable means. Sometimes warm applications increase the pain, cold then will generally relieve. When cold disagrees, warmth will relieve. I have known ice applied, and suspend the pain immediately, ly, but it again returned in a short period.

Gentle frictions with the hand, or some soft substance often soothes the acuteness of the pain, and renders it supportable. A gentleman from North Carolina, came to me labouring under a singular form of the disease. It alternated between the feet and the head. In the feet the pain was most acute. Frictions with the hand soothed its violence, and he had a servant whose sole occupation was to rub his master's feet. In an instant, the pain would cease in the feet and be transferred to the head. Here the feeling was as of an immense weight pressing and crushing down the head. His senses would become impaired, and he would continue repeating in rapid succession—"take that copper off my head"—struggling to relieve himself from his imagined burthen. This case was cured by large doses of quinia.

So embarrassing oftentimes is this class of affections, that it behoves us to have a vast number of resources at command. There is one mode of treatment which I have neglected to mention, which sometimes succeeds when all others have baffled us. It is the administration of arsenic. During the last summer, a very distressing case of neuralgia was relieved by Professor Chapman, on the exhibition of this remedy. A lady who had endured great mental distress was attacked with that form of the disease which I indicated to you, a violent pain at the extremity of the os cocygis. From this point, it irradiated up the whole spine, and thence over the body. The skin was so sensitive, that it was impossible to feel the pulse. All the usual remedies were tried without the slightest benefit. Day after day, day and night, was passed in agonising pain. Professor Chapman, who attended her with me, proposed trying Fowler's solution. It was accordingly done, and the dose augmented until a cholera morbus was induced. A large quantity of bilious matter, highly offensive to the smell, was ejected from the stomach, and the pain immediately ceased. Two weeks after, the symptoms re-appeared. The same mode of treatment was pursued with the same results, and permanent relief has continued since this last attack.

Sulphate of strychnia has also been recommended. This remedy acts on the spinal marrow, exciting the functions of the anterior columns, or segments of the spiral cord, and causing spasms. In his manner the posterior columns, or segments, may be relieved by a species of revulsion. I have twice made trial of it in bad cases. In one it entirely failed, after a long perseverance. In the other, partial relief was obtained. In both, the remedy was carried to the production of spasmodic contractions of the muscles.

---

*Saturday, February 3d.*—Dr. Harlan, whose tour of duty commenced this day, at 10 o'clock entered the lecture room and commenced:

Gentlemen:—In making my first appearance among you, in order to continue the clinical discourses to the end of the season, commenced by the Professor of Surgery in the University of Pennsylvania, I take occasion to remark, that the observations with which I shall occupy your attention will be entirely regulated by the nature of the cases which the wards of the hospital, consigned to my professional superintendence, present for treatment, believing as I do that, in a course of lectures strictly clinical, our remarks should be limited as far as possible to the history and treatment of the cases under immediate consideration.

Previously to entering upon the subjects of the present lecture, I beg leave to call your attention for a few moments, to the case of Clark, the coloured man, who, a few weeks since, was subjected to a most painful operation in the attempted removal of a tumour of the Antrum Highmorianum, implicating nearly the whole of the upper jaw. By the operator, my predecessor, you were duly informed at the time, of all the reasons which were supposed sufficiently urgent to authorize so important an undertaking. The fact that, as one of the consulting surgeons, I was opposed to the operation, was briefly stated. Under the conviction that the principle objects of a medical student are truth and knowledge, it becomes my duty to lay before you the arguments that induced me to oppose this, and which must influence me in all similar cases.

In the first place then, the extent, locality, and probable nature of the tumour appeared to me all in opposition to an operation. The tumour involved the antrum on both sides, and completely filled or obliterated the nasal cavities, causing also, extensive projections of the alveoli. Consequently a successful operation, or complete removal of the tumour, implied the destruction of the greater part of the face, especially of the organs of smell and of mastication, rendering life itself but a miserable boon, if finally preserved under such circumstances.

From the position of the tumour, such numerous and important arteries were involved, that the reiterated application of the actual cautery was rendered absolutely necessary, as the only means of obviating death from hemorrhage.

The nature of a tumour encased in bone, is of course, not so easily ascertained. From the history and progress of the disease, however, there was no reason to suspect it to be of a polypous nature, but the probability was in favour of its being of a more malignant nature, and involving more extensively the neighbouring tissues.

I had, further, seen two cases of a somewhat similar nature, but of less unpromising appearance, in which Drs. Physick and Parish were opposed to any surgical operation. From these, and other reasons, unnecessary now to detail, I was led to conclude that an operation in the present case was uncalled for, cruel in the first degree, as regards the patient's suffering, and opposed to the best interests of surgery, in as much as every operation unsuccessfully performed lessens the public estimation of the resources of our art, and deters remediable cases from timely application for surgical assistance.

To those members of the class who witnessed the operation, I need not dwell upon its appalling nature, the excruciating torments from red-hot pokers searing the lacerated throat, after the hammer and chissel had effected their purpose.

The sequence of the operation has more than confirmed my worst anticipations. I am unable to perceive the least degree of benefit resulting or possible to result from it. Unless, indeed, raising the facial angle, and approaching it to that of the Caucasian be thought a paramount improvement: an honour, which I suppose but few Ethiopians would have the ambition to attain by similar means.

The case, however, is yet open to your inspection, and if the patient be not cut off by some acute disease, a consummation rather desirable—the tumour alone will soon terminate his existence, as it appears to be already encroaching on the basis of the skull. The fatal termination of such cases is generally induced by pressure on the brain.

Dr. H. here commented on what he termed theatrical and medical surgery. Surgical operations, particularly amputation of limbs, formerly justly ranked among the *opprobria medicorum* were now comparatively rare, with the most enlightened members of the profession. Any eclát to be gained by mere manual dexterity is, said he, but a poor compensation for the sacrifice either of the interests of the profession or of the patient. One sure professional principle clearly illustrated or established, deserves better of mankind than all the mere operators put together.

Disclaiming all personal allusions, he read some extracts from a late number of the Medico Chirrug. Review, in an article by the editor of that journal, cautioning the pupil against any attempts at theatrical display in an art, which is daily becoming more of a science.

Numerous cases of inflammation of the eye, both acute and chronic, with some of the results of this diseased action were displayed to the class; their history and treatment commented upon, Dr. H. referring to the next lecture for a more detailed account of the methodus medendi, &c.

A remarkably fair case of cauliflower excrescence on the neck and lower jaw, involving the submental and maxillary glands, of several inches in diameter, was displayed. The chloride of zinc was prescribed as a caustic, and alteratives internally—subject to be resumed at next lecture.

---

*February* 10*th.*—At 10 o'clock Dr. Harlan commenced: I shall to day, gentlemen, offer a few remarks upon some of the varieties of Ophthalmia. I shall confine my observations to the cases in the house, and thus render my lecture as much as practicable, purely clinical. I shall first speak of purulent ophthalma; this has been called also Egyptian ophthalmia, from the great frequency of its occurrence in that country. It is a very violent and intractable affection, ending sometimes in disorganization of the eyes. The French army under Napoleon, when in Egypt, suffered very much from it. The causes of this form of ophthalmia are not all well ascertained. By some it has been attributed to the sand; small particles of fine sand get into the eye, irritating it and producing violent inflammation. This disease is not confined to Egypt, and prevails in other countries, where the same cause does not obtain. Larrey in his surgery makes a remark, which seems to me to bear on this subject. He says that those soldiers who slept with night caps drawn over their eyes, escaped the affection. It is said that in those countries where purulent ophthalmia is endemic, that the inhabitants who dwell in closely built houses are exempt. From these facts I am inclined to believe that purulent or Egyptian ophthalmia is caused by absence of moisture in that country. A well attested fact in natural history, adds weight to this opinion; the camel, previous to sleeping at night, scrapes a hole in the sand, over which he lodges his head, which is thus exposed to the exhalations of moisture from the earth. This animal is known never to suffer from purulent ophthalmia. Just at this moment we do not happen to have many cases in the house, although it is not by any means an uncommon affection here.

It requires the most active treatment. The mucous membrane lining the eyelids and ball, is in a state of high and violent inflammation, which extends to the cornea, causing it to slough, and ends in the disorganization of the eye, and consequent loss of sight. The lids occasionally, are agglutinated together, and slough occurs within.

I think of all the methods of treatment, that by brisk purgatives is most to be relied on. Those which I prefer are the saline cathartics: keep up a constant drain from the bowels with these for the space of several days, and you will derive considerable benefit. To the use of purgatives must be conjoined more or less extensively, that of general and local bleeding.

[Dr. H. here exhibited several patients to the class.]

This, like all other inflammations, may arise from a local cause, and be connected with, and modified by a specific disease, as scrofula, lues venerea, &c. The purulent ophthalmia of negroes is almost always scrofulous.

After active depletion and purging we may have recourse to mild astringents, and the best of these is the sulphate of zinc. Zinc exercises a very peculiar effect on the mucous membranes, and may be used when no other astringent is admissable. In gonorrhea it may be employed during the existence of the ardor urinæ, giving ease and comfort to the patient. I use it combined with Armenian Bole and opium.

In venereal opthalmia, where the conjunctiva are gorged with blood, I have found a strong solution of the nitrate of silver (15 grs. to aq. dist. ſſ℥i) to be of great service.

Adhesions sometimes occur between the iris and the adjacent tunics, constituting Synechia,—anterior or posterior, as the attachment of the iris is to the cornea or to the Leris. In such cases we employ the extract of stramonium, which by causing dilatation of the pupil breaks up the connections. An alum curd is very often used with advantage in chronic ophthalmia. It is made by the coagulation of milk by alum, and applied to the eye for several hours, a piece of gauze intervening between the eye and curd.

This man came into the house with acute inflammation of the eyes and extensive ulcerations on the cornea. He is now suffering from entropion, not an uncommon result of external chronic inflammation. A contraction and twist of the ciliary margins take place, and the edge of the lid is inverted, and the eye-lashes being brought constantly against the cornea, produce and keep up irritation. There have been various methods proposed to cure this disagreeable and troublesome deformity. Some cut a triangular piece out of the lid, and bring the edges of the wound together. I prefer to touch the exterior of the lid with a piece of red hot iron wire. The great contraction of the cicatrix of a burn is well known, and it is in this way a cure is effected.

The next case that I show you is a man who has just come into the house with fracture of the ribs. A spicula of bone has been driven in through the pleura, wounding the lung, and causing an escape of air into the cellular tissue of the body, and producing that condition called emphysema. You perceive that the swelling is not confined to one spot, but that it has diffused itself over the whole chest, and is extending throughout the body generally. I wish you to examine, after lecture, this patient particularly. The sensation communicated to the touch is peculiar and will not readily be forgotten. The sound which we hear on putting the ear close to the skin is crackling, and has improperly been styled a crepitus.

Hucksters are very much in the habit of making their poultry appear fat, by blowing up the cellular structure. Lean turkeys thus are made to assume the appearance of great plumpness.

Both surfaces of the pleura—the pleura costalis and the pleura pulmonalis, are wounded in this accident, and an artificial, or mechanical pleurisy is induced, and we are usually obliged to resort to bloodletting. In this case there is exhaustion of the system; the circulation is very feeble, the skin is, as you perceive livid, from retarded circulation.

I shall to this man's chest apply a bandage for a two fold reason—first to produce an absorption of the air lodged in the cellular tissue; and secondly, to prevent any action of the muscles of the chest, to paralyse these, and cause the patient to breathe by the diaphragm solely. I shall direct to be administered to him also, some carbonate of ammonia, as a gentle and excellent stimulant, his pulse being below par.

We had a similar case last week in the house which terminated fatally from serous effusion. The man had been bled very copiously before entering. This is often a very common cause of death in such cases, occurring in worn constitutions.

I now exhibit to you a specimen of cauliflower ulcer of the throat and lower jaw. I have applied to this a caustic composed of flour and chloride of zinc. This is a most excellent caustic application, and one which I frequently employ. It does not spread to the sound parts; it eats downwards and not laterally. It also possesses the power of coagulating the blood in the vessels, and prevents hermorhage. A weak solution in water, is a good application to indolent ulcers, and you can apply it just as you would nitric acid, and on the same principle. You can make it of any strength. The usual strength is two parts flour to one of the zinc; it can be left on from twenty-four hours, to three days.

In one case in which I used it for the removal of a cancerous tumour in the axilla, it was followed by unpleasant consequences. The woman had a large open cancer of the breast alvo, the caustic was applied to the axillary tumour and a profuse hermorrhage occurred from a large vein.

The object of flour is to weaken the caustic, and also to give it consistence. The chloride of zinc is a very deliquescent salt. It does not act powerfully on the skin. I frequently make it up with my fingers without their suffering. Indeed, in cases where the integrity of the skin exists, you must first apply a blister, and remove the cuticle, before the caustic will act. The application may be made of any thickness, proportionate to the effect to be produced, from one to five lines. Within the last twelve months I have resorted very frequently to this caustic, for the removal of cancerous ulcers and schirrhus, agreeably to the mode detailed by Dr. Canquoin in his memoir on this subject, but regret to say, not by any means with the success which his statements led us to anticipate. The same results occurred in the hands of Dr. Ashmead, who used this caustic very extensively. In most, if not all of his cancerous cases, in which this caustic was applied, the disease returned.

